# A Paradox Unveiled: A Case Report of Cerebral Infarctions in a Patient With Severe Thrombocytopenia

**DOI:** 10.7759/cureus.65283

**Published:** 2024-07-24

**Authors:** Volha Chapiolkina, Yemesrach F Mekonen, Nehemias Guevara, Esmirna Perez, Jorge Sanchez, Maria C Tole, Ivette Vigoda

**Affiliations:** 1 Internal Medicine, St. Barnabas Hospital Health System, New York, USA; 2 Medicine, St. Barnabas Hospital Health System, New York, USA; 3 Oncology, St. Barnabas Hospital Health System, New York, USA

**Keywords:** treatment, platelet count, idiopathic thrombocytopenic purpura (itp), cerebral infarction, ischemic stroke

## Abstract

Thrombocytopenia is a condition in which the platelet count is less than 150,000/μL, which can be congenital or acquired. The condition can be further sub-classified. Nevertheless, the causes include infection, medication-mediated, liver diseases, or heart diseases. Moreover, diagnosis is straightforward only on a few occasions. Here, we are presenting a patient with a conundrum of immune thrombocytopenia (ITP) and a stroke. A 75-year-old female patient with a past medical history of hypertension was brought to the emergency department (ED) for altered mental status (AMS). Initial blood workup showed a platelet count of 27,000/μL and hemoglobin level of 6.2 g/dl, and brain magnetic resonance imaging (MRI) revealed ischemic stroke. Rarely, ITP patients can paradoxically develop arterial and venous thrombosis. Hence, physicians must remain vigilant in promptly and accurately diagnosing thrombotic events in ITP to ensure appropriate treatment, including antiplatelet and anticoagulant therapy, alongside ITP-specific interventions to improve outcomes.

## Introduction

Immune thrombocytopenia (ITP) is an acquired thrombocytopenia caused by autoantibodies against platelet antigens [1]. Weycker et al. report that ITP incidence in the United States is 6.1 per 100,000 persons, with a higher incidence of 6.7 per 100,000 among females [2]. A considerable number of ITP patients do not exhibit any symptoms or show only mild mucocutaneous hemorrhage. However, 5-6% experience severe bleeding [3]. A 2014 study found that bleeding episodes, infections, and cardiovascular events in ITP patients resulted in 1.3-2.2 times higher mortality rates than the general population [4]. Individuals with ITP have a relatively small but significant risk of developing thromboembolism, as demonstrated by a meta-analysis conducted by Doobaree et al. The study found that ITP patients have an increased risk of arterial thromboembolism (ATE) and a higher risk of venous thromboembolism (VTE), and the risk increases in patients with splenectomy [5]. It has also been reported that older age is associated with thrombosis in ITP patients [6]. Various factors contribute to thrombosis in patients with ITP. These include ITP treatment, other health conditions that may be present, complement activation, endothelial activation, inhibition of natural anticoagulants, young hyperactive platelets, platelet microparticles, and a rebalanced hemostasis. All these factors play a role in the pathophysiology of thrombosis in ITP [7]. ​Here, we report a case of a 75-year-old female patient presenting with severe thrombocytopenia and multifocal ischemic infarcts.

## Case presentation

A 75-year-old female with a past medical history of hypertension was brought to the emergency department (ED) for altered mental status. As per the family, the patient had been weak for two days and barely got up from the chair. On the day of admission, the patient stopped communicating, which led the husband to call emergency medical services. As per the family, she occasionally drinks wine and denied cigarette smoking, pet exposure, recent travel, or traumatic injury. On physical examination (PE), the patient was lethargic and had a heart rate of 96 per minute, a temperature of 36.2°C, a blood pressure of 107/69 mmHg, and a respiratory rate of 20 per minute; she had pale conjunctiva, multiple bruises on the bilateral upper and lower extremities with a Glasgow coma score of 10/15 and right facial droop, but moved all extremities away from noxious stimuli and Brudzinski and Kernick signs were negative. 

Initial blood workup showed a platelet count of 27,000/μL and hemoglobin level of 6.2 g/dl, hypoalbuminemia, acute kidney injury, in addition to elevated lactate dehydrogenase (LDH), C-reactive protein (CRP), and sedimentation rate (ESR), and low fibrinogen (Table 1). Due to altered mental status, a CT scan of the brain was performed in the ED, and it showed no acute pathology. She received one unit of blood.

**Table 1:** Table 1. Blood workup on admission MCV: Mean corpuscular volume; ALT: alanine aminotransferase; AST: aspartate aminotransferase; INR: international normalized ratio; TSH: thyroid stimulating hormone

Variable	On admission	Reference range
White blood cell count	15	4.2-9.1 x10^3^/μL
Hemoglobin	6.2	13.7-17.5 gm/dL
Hematocrit	16.5	40.1-51.0%
MCV	83.3	79.0-92.2 fL
Platelet count	27	150-450x 10^3^/μL
ALT	10	4-36 IU/L
AST	13	8-33 IU/L
Alkaline phosphatase	51	38-126 IU/L
Bilirubin total	0.4	0.1-1.2 mg/dL
INR	1.09	0.9-1.1
Calcium	8.4	9.2-11.0 mg/dL
Lactic acid	0.4	0.5-2.2 mmol/l
Albumin	2.4	3.8-5.0 gm/dL

**Figure 1:**
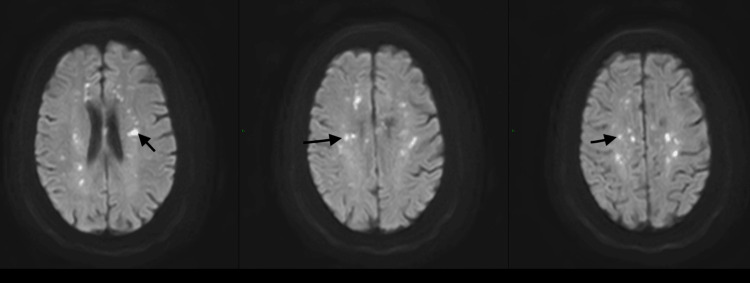
MRI showing extensive bilateral subacute cerebral infarcts (arrows).

**Figure 2:**
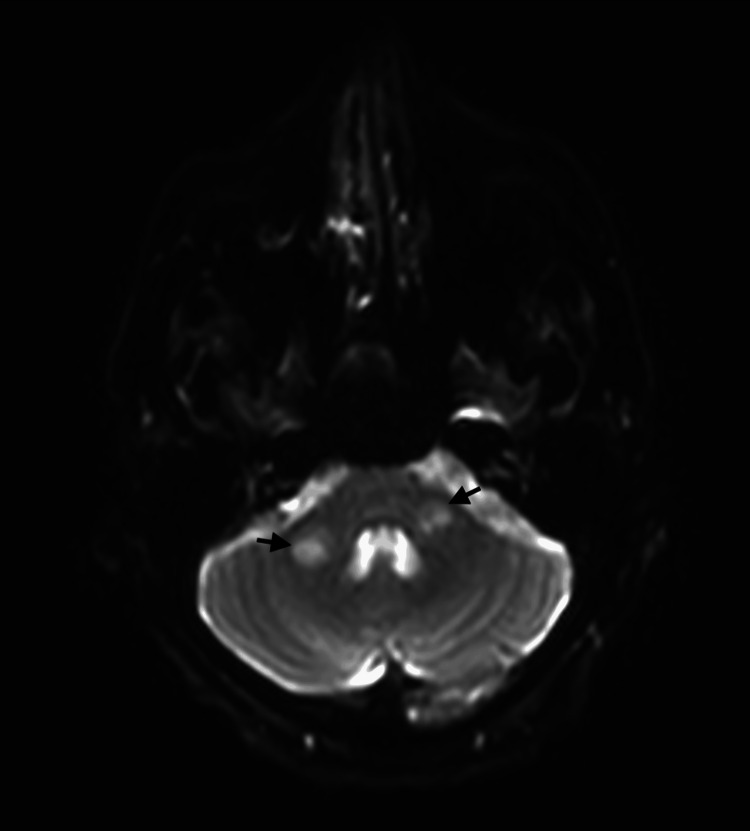
MRI showing subacute brainstem and middle cerebellar peduncle infarcts (arrows).

However, due to a high suspicion of stroke based on the physical examination, brain magnetic resonance imaging (MRI) was performed (Figures 1, 2), and multiple infarcts in bilateral cerebral hemispheres and brainstem were revealed. Further workup revealed persistent thrombocytopenia, which was proven with a peripheral smear, without hemolysis.

**Figure 3:**
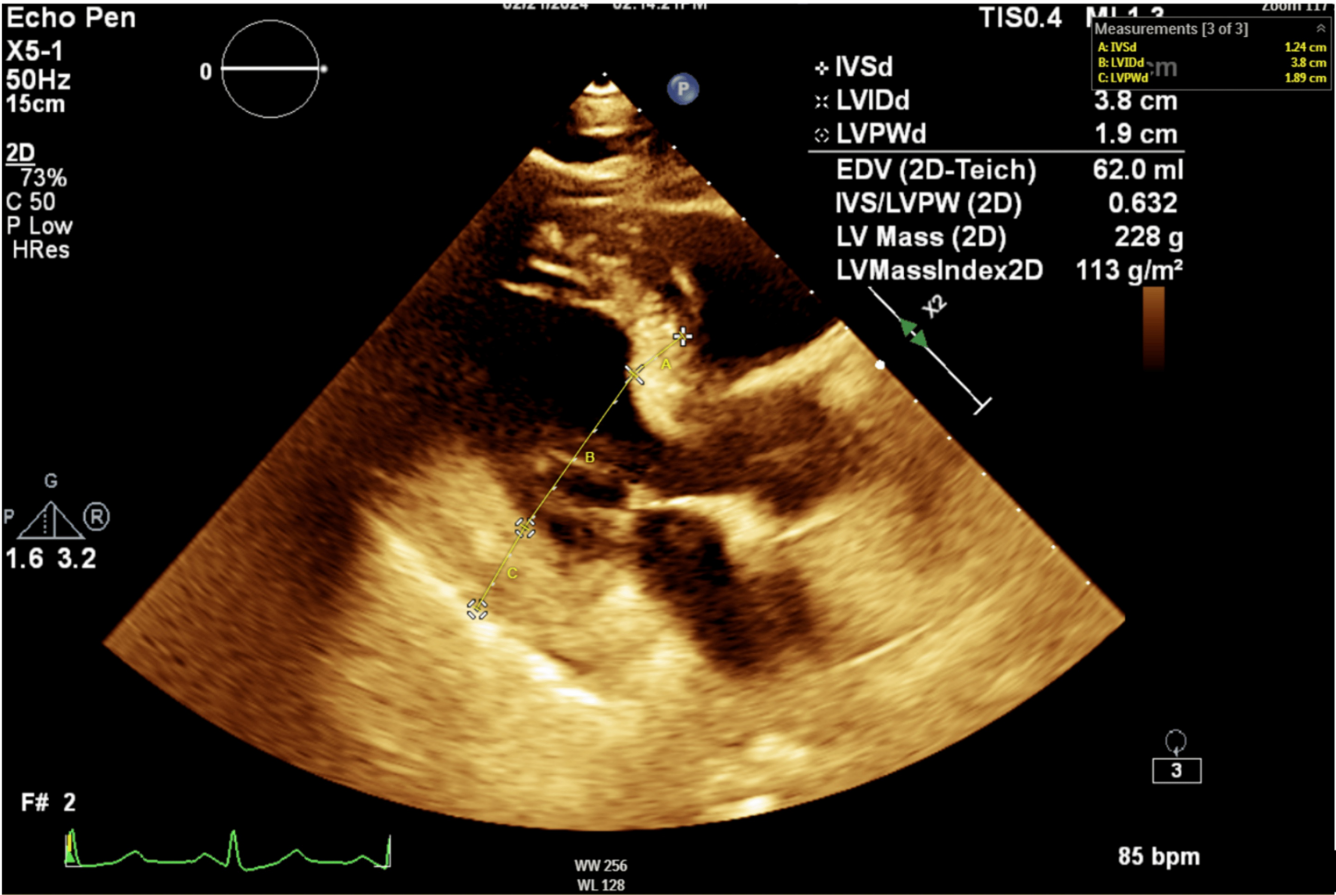
Transthoracic echocardiography parasternal long axis view showing mild left ventricular hypertrophy.

**Figure 4:**
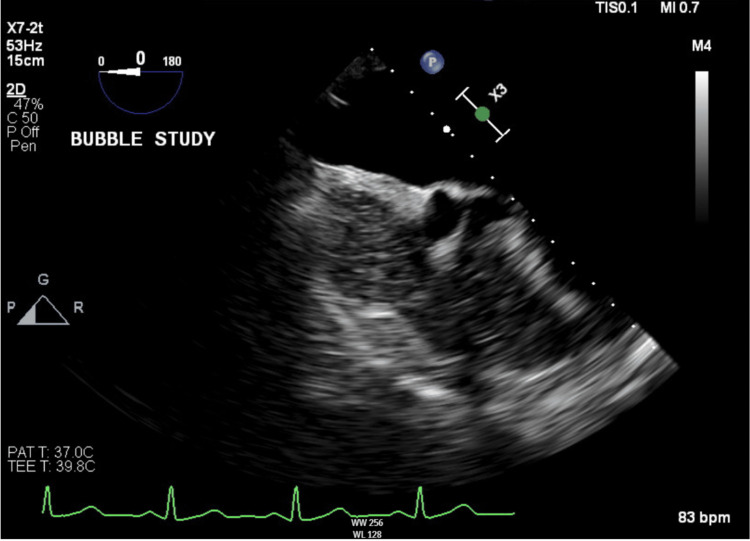
Normal transesophageal echocardiography with bubble study.

Because of the brain MRI findings, transthoracic echocardiography (TTE) was performed, which showed mild left ventricular hypertrophy, grade one diastolic dysfunction, and normal ejection fraction (Figure 3). The Holter monitor showed a sinus rhythm. In addition, TTE with bubble study was done, which was normal (Figure 4). The Holter monitor showed sinus rhythm.

A hematology service was consulted, which recommended bone marrow biopsy and additional laboratory studies (Table 2). The bone marrow biopsy showed normocellular marrow with 35-45% cellularity, trilineage hematopoiesis, and maturation. There was no immunophenotypic evidence of abnormal myeloid maturation, increased blast population, or a lymphoproliferative disorder.



**Table 2:** Blood workup after admission AB: Antibody; ADAMTS: A disintegrin and metalloproteinase with thrombospondin type 1 repeats, member 13; IgG: immunoglobulin G; TIBC: total iron-binding capacity; TSH: thyroid stimulating hormone; GPL: IgG phospholipid units.

Variable	After admission	Reference
Iron	54	50-170 μg/dL
Ferritin	25	12-150 ng/mL
Iron Saturation	6.7	20-55%
TIBC	239	250-400 μg/dl
Vitamin b12	176	160-950 pg/ml
Folate	9.9	>3n g/ml
Homocysteine	10.3	0-19 μmol/l
D-dimer	2.25	<0.5 mg/dl
Fibrinogen	165	200-450 mg/dl
Lupus anticoagulant	43	0-47 sec
Cardiolipin IGG	<9	<9 GPL u/ml
Beta 2 glycoprotein IGG	<9	<9 GPL u/ml
Immunofixation	Negative	Negative
M spike	Negative	Negative
TSH	1.26	0.45-5.33 IU/ml
ADAMTS 13 AB	8	<12 units/ml

The patient's platelet count further decreased to 6000/μl, and patient was transfused with one unit of platelets. Then the platelet count increased to 37,000 and she was started on dexamethasone 40mg intravenous every 24 hours for four days with the consideration of ITP. Subsequently, the platelet count increased to 101,000/μl within four days of steroid administration; afterward, she was started on aspirin. The patient's mental status and facial droop improved, but she had dysarthria. She was discharged with a prescription of aspirin, atorvastatin, and lisinopril. Oral steroid was not started because the patient had responded well to pulse dexamethasone. During the neurology outpatient clinic visit, the patient's overall condition was better, and she had no focal deficit besides dysarthria. Her last platelet count was 206,000/μl. Currently, the patient is pending hematology-oncology and cardiology outpatient follow-ups. 

## Discussion

Primary or secondary ITP has historically been known as an acquired bleeding disorder characterized by autoantibodies against platelets, which results in platelet destruction or underproduction [1]. The incidence of immune thrombocytopenic purpura in the general population is roughly estimated to be between two and five cases per 100,000 people [2]. ITP has variable clinical presentation, with thrombocytopenia and bleeding being the common symptoms. However, some cases reported only some nonspecific symptoms, such as fatigue or being completely asymptomatic [8]. The lack of a sensitive or specific diagnostic tool for ITP and the variety of reasons for other potential causes of low platelet numbers, some of which may be overlooked (e.g., liver disease, drug-induced thrombocytopenia, hereditary platelet disorder), contribute to the challenges in diagnosing ITP [9].

ITP is an autoimmune condition in which the thrombocytes and their precursors become targets of a malfunctioning immune system. Most autoantibodies in patients with ITP belong to the immunoglobulin 1 (IgG1) subclass and are potent classical complement pathway activators [10]. Antibody-coated platelets can initiate complement activation via the classical pathway that initiates direct platelet destruction and increases the clearance of C3b-coated platelets by complement receptors. Similar autoantibody interactions with bone marrow naive thrombocytes can also result in complement injury and ineffective thrombopoiesis [10]. This synergy causes a decrease in platelet number. Eventually, it leads to a bleeding event that can present with hemorrhages in the skin, on the mucous membrane, or even intracranial hemorrhagic events [10]. 

Patients with ITP usually have an extremely low platelet count that commonly causes hemorrhagic complications, and they occasionally experience ischemic stroke. One of the unusual ITP presentations is paradoxical thrombosis [8]. Thrombus formation in ITP is related to the pathophysiology of the disease (naive hyperactive thrombocytes, platelet microparticles, impaired hemostasis, activation and damage of endothelium, antiphospholipid antibodies, and inhibition of natural anticoagulants), ITP treatment, and other comorbidities that altogether contribute to the incidence of thrombosis. The most recent studies suggest multifactorial pathogenesis, with platelet microparticles (PMPs) playing the primary role along with other understudied mechanisms in vascular cell activation and endothelial wall damage [11].

Other contributing factors are the formation of platelet-leukocyte-monocyte aggregates, endothelium-activating antibodies, complement system activation, and low levels of A disintegrin and metalloproteinase with thrombospondin type 1 repeats, member 13 (ADAMTS-13) [12]. Moreover, epidemiological studies have demonstrated a higher risk of venous thromboembolism in ITP patients compared to patients with arterial thromboembolism. Nevertheless, there are conflicting findings regarding the risk of specific arterial events, such as myocardial infarction and ischemic stroke, in our case. Additionally, a subset of ITP patients with elevated platelet activation markers may be at increased risk of vascular dementia as well [13]. Exposure to phospholipase A2 releases arachidonic acid, metabolized by the platelet to thromboxane A2, a severe procoagulant [14]. The risk of thrombosis in ITP could be higher than expected, making the management and diagnosis of ITP more complicated. Therefore, an extensive workup, like in our case, should be performed. The paradoxical mechanism of ITP-associated thrombus formation requires further exploration and research.

Treatment of stroke/paradoxical thromboembolism in the setting of ITP is still a challenge and requires careful consideration of thrombolysis, antiplatelet agents, and anticoagulants [15]. Thrombolysis with tissue plasminogen activator (tPA) is generally contraindicated in patients with platelet counts below 100 × 10^5^/L due to bleeding risks. However, there are limited reports of successful intraarterial thrombolysis in ITP patients with adequate platelet counts [16]. The safety and efficacy of tPA in ITP patients remain unknown; therefore, further research is needed in this area. Antiplatelet agents and anticoagulants may be cautiously used based on individual patient factors and risk assessment, although their effects on ITP patients are not fully understood [17].

On the other hand, combination therapy with immunosuppressants, such as corticosteroids and intravenous immunoglobulin (IVIg), followed by antiplatelet agents, shows promise but requires further investigation [17]. However, each patient must be thoroughly evaluated, outweighing the risks and benefits before initiating treatment. In our patient, she was discharged with aspirin and atorvastatin.

## Conclusions

Our case underscores the critical need for heightened vigilance among physicians when encountering patients with severe thrombocytopenia, as thromboembolic events can occur as an initial presentation of immune thrombocytopenic purpura. Despite the traditionally hemorrhagic nature of ITP, the risk of paradoxical thrombosis remains significant and necessitates an extensive diagnostic evaluation. Furthermore, early recognition and accurate diagnosis of thrombotic complications in ITP are imperative to initiate timely and appropriate treatments, such as antiplatelet and anticoagulant therapies, in conjunction with ITP-specific interventions. This approach is essential to mitigate risks and enhance patient outcomes, highlighting the complex interplay between thrombosis and thrombocytopenia in ITP and the importance of a multidisciplinary approach to management.

## References

[1] Cines DB, Blanchette VS (2002). Immune thrombocytopenic purpura. N Engl J Med.

[2] Weycker D, Hanau A, Hatfield M (2020). Primary immune thrombocytopenia in US clinical practice: incidence and healthcare burden in first 12 months following diagnosis. J Med Econ.

[3] Piel-Julian ML, Mahévas M, Germain J (2018). Risk factors for bleeding, including platelet count threshold, in newly diagnosed immune thrombocytopenia adults. J Thromb Haemost.

[4] Frederiksen H, Maegbaek ML, Nørgaard M (2014). Twenty-year mortality of adult patients with primary immune thrombocytopenia: a Danish population-based cohort study. Br J Haematol.

[5] Doobaree IU, Nandigam R, Bennett D, Newland A, Provan D (2016). Thromboembolism in adults with primary immune thrombocytopenia: a systematic literature review and meta-analysis. Eur J Haematol.

[6] Ruggeri M, Tosetto A, Palandri F (2014). Thrombotic risk in patients with primary immune thrombocytopenia is only mildly increased and explained by personal and treatment-related risk factors. J Thromb Haemost.

[7] Tărniceriu CC, Hurjui LL, Florea ID (2022). Immune thrombocytopenic purpura as a hemorrhagic versus thrombotic disease: an updated insight into pathophysiological mechanisms. Medicina (Kaunas).

[8] Cines DB, Bussel JB, Liebman HA, Luning Prak ET (2009). The ITP syndrome: pathogenic and clinical diversity. Blood.

[9] Kohli R, Chaturvedi S (2019). Epidemiology and clinical manifestations of immune thrombocytopenia. Hamostaseologie.

[10] Weitz IC, Liebman HA (2023). Complement in immune thrombocytopenia (ITP): the role of complement in refractory ITP. Br J Haematol.

[11] Owens AP, Mackman N (2011). Microparticles in hemostasis and thrombosis. Circ Res.

[12] Sarpatwari A, Bennett D, Logie JW (2010). Thromboembolic events among adult patients with primary immune thrombocytopenia in the United Kingdom General Practice Research Database. Haematologica.

[13] Enger C, Bennett D, Forssen U, Fogarty PF, McAfee AT (2010). Comorbidities in patients with persistent or chronic immune thrombocytopenia. Int J Hematol.

[14] Italiano JE Jr, Mairuhu AT, Flaumenhaft R (2010). Clinical relevance of microparticles from platelets and megakaryocytes. Curr Opin Hematol.

[15] Pishko AM, Misgav M, Cuker A, Cines DB, George JN, Vesely SK, Terrell DR (2018). Management of antithrombotic therapy in adults with immune thrombocytopenia (ITP): a survey of ITP specialists and general hematologist-oncologists. J Thromb Thrombolysis.

[16] Alrohimi A, Purdy K, Alqarni M (2021). The clinical conundrum of managing ischemic stroke in patients with immune thrombocytopenia. Can J Neurol Sci.

[17] Mowla A, Kamal H, Lail NS (2017). Intravenous thrombolysis for acute ischemic stroke in patients with thrombocytopenia. J Stroke Cerebrovasc Dis.

